# Growth and nutritional status according to the number of sensitized food allergens in infants and young children with atopic dermatitis

**DOI:** 10.1186/2045-7022-1-S1-P59

**Published:** 2011-08-12

**Authors:** Hye Yung Yum

**Affiliations:** 1 Seoul Medical Center, Atopy Clinic, Seoul, Republic of Korea

## Background

Food allergy could affect the growth and nutritional status of children with atopic dermatitis (AD). This study was conducted to determine the association of the number of sensitized food allergens with growth and nutritional status in infants and young children with AD.

## Methods

We studied 165 children with AD aged 5 to 47 months with AD, who visited the Atopy Clinic of Seoul Medical Center. We checked birth weight, time of starting weaning foods, severity scores of atopic dermatitis (SCORAD), eosinophil counts in peripheral blood, serum total IgE and specific IgE to 6 major allergens (egg white, cow's milk, soybean, peanut, wheat and fish). Height and weight for age and weight for height were converted to Z-scores to evaluate their effects on growth and nutritional status. Specific IgE levels ≥ 0.7 kUA/L by the CAP assay were considered positive.

## Results

As the number of sensitized food allergens increased, the mean Z-scores of weight and height-for-age were decreased (P = 0.006 and 0.018, respectively). This number was directly correlated with SCORAD (r = 0.308), time of starting weaning foods (r = 0.332), eosinophil counts in peripheral blood (r = 0.266) and serum total IgE (r = 0.394), while it was inversely correlated with the Z-scores of weight for age (r = -0.358), height for age (r = -0.278) and weight for height (r = -0.224).

## Conclusions

The increased number of sensitized food allergens had a negative effect on growth and nutritional status in infants and young children with AD. Therefore, a thorough evaluation of growth and nutritional status and adequate management are crucial in pediatric AD patients with a larger number of sensitized food allergens.

